# 358. Early Cardiac Marker of Mortality in COVID-19

**DOI:** 10.1093/ofid/ofab466.559

**Published:** 2021-12-04

**Authors:** Douglas Salguero, Juliana Ferri-Guerra, Angel Porras, Marissa Donatelle, Everett Rogers, Lee Seiffer, Ruben Porudominsky, francisco Ujueta, Ayoola Olayiwola, Claudio Tuda

**Affiliations:** Mount Sinai Medical Center, Miami, Florida

## Abstract

**Background:**

Epicardial adipose tissue (EAT) is a highly inflammatory depot of fat, with high concentrations of IL-6 and macrophages, which can directly reach the myo-pericardium via the vasa vasorum or paracrine pathways. TNF-α and IL-6 diminish cardiac inotropic function, making EAT inflammation a potential cause of cardiac dysfunction.

**Methods:**

A retrospective cohort study assessing EAT Thickness and Density from CT scans, without contrast, from adult patients during index admission for COVID-19 infection at Mount Sinai Medical Center from March 2020 to January 2021. A total of 1,644 patients were screened, of which 148 patients were included. Follow-up completed until death or discharge. The descriptive analysis was applied to the general population, parametric test of normality for comparisons between groups. Kaplan survival analysis was conducted after survival distribution was confirmed significant. It was followed by the assumption of normality by Q-Q Plot, prior to performing a multiple regression analysis in the vulnerable group using a K-Matrix input for cofounders. A log-rank test was conducted to determine differences in the survival distributions for the different ranges of EAT thickness.

**Results:**

A total of 148 Participants were assigned to two groups based on epicardial adipose tissue in order to classify them as increased or decreased risk of cardiovascular risk: >5mm (n = 99), < 5mm (n = 49). The survival percentage was higher in the group with no EAT inflammation compared to the group with EAT inflammation (95.0% and 65%, respectively). Participants with EAT >5mm had a median day of hospital stay of 18 (95% CI, 16.86 to 29.92). The survival distributions for the two categories were statistically significantly different, χ2(2) = 6.9, p < 0.01. A Bonferroni correction was made with statistical significance accepted at the p < 0.025 level. There was a statistically significant difference in survival distributions for the EAT >5 mm vs EAT < 5 mm, χ2(1) =6.953, p = 0.008.

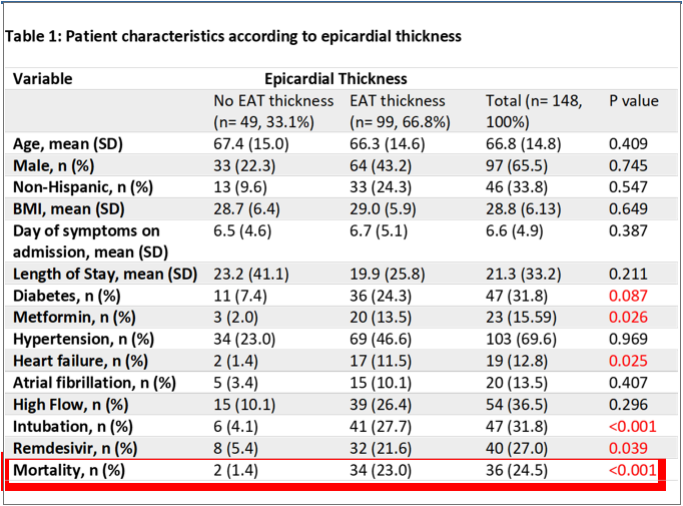

EAT Thickness Survival Analysis 2020-2021 COVID-19 MSMC

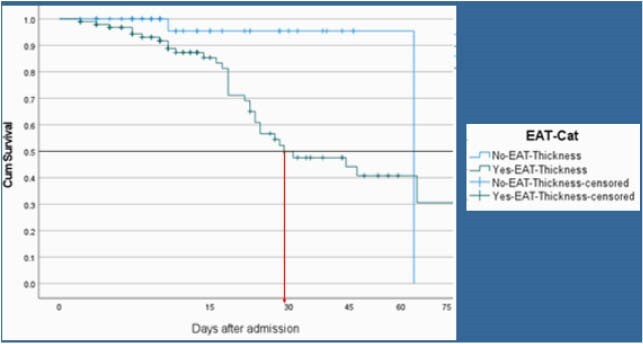

Scatter Plot Length of Stay by EAT Thickness

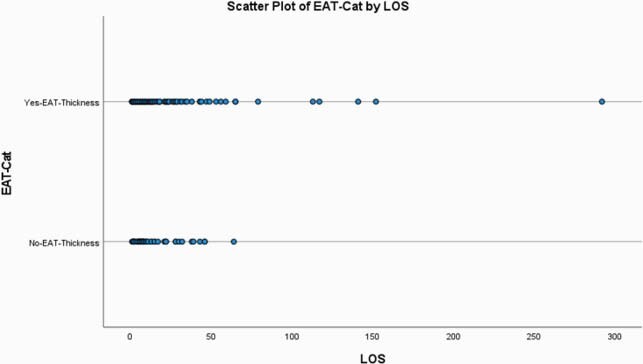

**Conclusion:**

There was an association with increased EAT thickness and increased mortality. These findings suggest that EAT thickness can be used as a prognostic factor and as a risk factor for increased mortality in patients with COVID-19

**Disclosures:**

**All Authors**: No reported disclosures

